# Systematic review and rationale of using psychedelics in the treatment of cannabis use disorder

**DOI:** 10.3389/fpsyt.2023.1144276

**Published:** 2023-06-26

**Authors:** Angela N. Phan, Garth E. Terry

**Affiliations:** ^1^University of Washington School of Medicine, Seattle, WA, United States; ^2^Departments of Psychiatry and Behavioral Sciences, and Radiology, University of Washington School of Medicine, Seattle, WA, United States; ^3^Mental Illness Research, Education, and Clinical Center (MIRECC), Department of Veterans Affairs, Puget Sound Health Care System, Seattle, WA, United States

**Keywords:** cannabis use disorder, psychedelics, psilocybin, MDMA, ketamine

## Abstract

**Introduction:**

Cannabis use disorder (CUD) is prevalent in ~2–5% of adults in the United States and is anticipated to increase as restrictions to cannabis decrease and tetrahydrocannabinol (THC) content in cannabis products increase. No FDA-approved medications for CUD are currently available, despite trials of dozens of re-purposed and novel drugs. Psychedelics have garnered interest as a therapeutic class in other substance use disorders, and self-report surveys suggest they may result in positive outcomes for CUD. Herein, we review the existing literature pertaining to psychedelic use in persons with or at risk for CUD and consider the potential rationale underpinning psychedelics as a treatment for CUD.

**Methods:**

A systematic search was performed in several databases. Inclusion criteria were primary research reporting use of psychedelics or related substances and CUD for treatment in human subjects. Exclusion criteria were results including psychedelics or related substances without changes in cannabis use or risks associated with CUD.

**Results:**

Three hundred and five unique results were returned. One article was identified using the non-classical psychedelic ketamine in CUD; three articles were identified as topically relevant based on their secondary data or consideration of mechanism. Additional articles were reviewed for purposes of background, review of safety considerations, and formulating rationale.

**Conclusion:**

Limited data and reporting are available on the use of psychedelics in persons with CUD, and more research is needed given the anticipated increase in CUD incidence and increasing interest in psychedelic use. While psychedelics, broadly, have a high therapeutic index with infrequent serious adverse effects, particular adverse effects at risk in the CUD population, such as psychosis and cardiovascular events, should be considered. Possible mechanisms by which psychedelics have therapeutic potential in CUD are explored.

## Introduction

Cannabis (aka, marijuana) is the most commonly used illicit psychoactive substance and third overall after alcohol and tobacco. As state laws become more permissive of cannabis use and attitudes toward cannabis become more accepting with less perception of risk over time, cannabis use disorder (CUD) will likely become more prevalent. About 9% of those who ever use cannabis and 50% those who use cannabis daily will develop CUD over their lifetime (1, 2). Recently, it has been estimated that ~5% of those 12 or older in the United States met criteria for CUD in the past year (3). Although cannabis has increasingly been sought to treat conditions including nausea, pain, and psychiatric disorders including anxiety, those seeking to use cannabis for medical benefits are also at increased risk for developing CUD (4).

Similar to other substance use disorders (SUDs), CUD is defined with symptoms including: persistent desire or craving to use cannabis; unsuccessful efforts to cut down or control use; spending a great deal of time to obtain, use, or recover from cannabis; reducing social, occupational, or recreational obligations due to cannabis use; among other criteria (5). By definition, those with CUD have significant impairment or distress resulting from cannabis use. As a result, individuals with CUD may experience difficulty achieving milestones and have deficits in multiple domains of functioning. As in other SUDs, abruptly stopping cannabis can lead to withdrawal symptoms, adding to the difficulty of a person's ability to quit (6). A particular complication of chronic cannabis use is development of cannabis hyperemesis syndrome, which is defined as cyclic bouts of nausea and vomiting (to a potentially life-threatening degree due to dehydration and electrolyte imbalance) that are characteristically alleviated by hot showers or baths, may be unresponsive to typical antiemetics, and resolve with sustained cannabis cessation.

Importantly, the amount of tetrahydrocannabinol (THC) in cannabis continues to increase over time both according to DEA drug seizures (7) and as observed in cannabis markets (8). THC is the primary compound in cannabis that is responsible for its psychoactive effects, and higher concentrations of THC (sometimes referred to as potency) in grown cannabis and prepared cannabis products are associated with more negative outcomes. For example, THC is dose-dependently correlated to the development of CUD symptom onset, psychomotor impairment, risk for motor vehicle accidents, and prolonged increase of heart rate (9–11).

Despite the access and use of cannabis becoming more widespread, it is not without adverse consequences. Cannabis has been linked to worsening the symptoms and course of psychiatric disorders, such as psychosis and schizophrenia (12), associated with more poor outcomes in mood and anxiety disorders (13, 14), and adversely affecting cognitive functioning (15). Cannabis smokers have been shown to have with pulmonary findings consistent with chronic bronchitis and emphysema (16). Following cannabis use, whether smoked or not, individuals may experience increased cardiac work load including elevated heart rate, vasospasm, and increased myocardial oxygen demand—all increasing the risk of cardio- and cerebrovascular events (17).

Interestingly, the neurobiology of cannabis addiction has some unique differences from the classic model established for other SUD and behavioral addictions. THC has been shown to be reinforcing in humans and is likely responsible for driving tolerance and dependence of cannabis (18), but animal models of self-administration of THC, while possible, have been more challenging to develop than those for stimulants, opioids, nicotine, or alcohol (19). While THC has been shown to increase dopamine release in the nucleus accumbens in animals, positron emission tomography (PET) studies in humans have neither consistently nor robustly reflected this result (20, 21), while imaging reduced dopamine synthesis in the prefrontal cortex correlated to symptoms of apathy in CUD patients (22). Furthermore, unlike other substances of abuse, individuals with chronic cannabis use do not exhibit altered D_2/3_ receptor availability in striatum (23). This lends intrigue to the possibility that the neurobiology of CUD may be driven by elements of the addiction neurocircuitry outside of dopaminergic modulation within the nucleus accumbens.

The endocannabinoid system (ECS) has a crucial role in the neuromodulation of rewarding and neurophysiological actions by drugs of abuse. A suite of lipid neurotransmitters, their synthetic and catabolic enzymes, and their preferential cannabinoid receptors (CB_1_, CB_2_), the ECS modulates release of numerous other neurotransmitters and therefore offers a key contribution to learning and behavioral responses. CB_1_ receptors are expressed throughout the brain and with high density, particularly in brain regions mediating addiction, and by co-localizing with GABAergic interneurons and glutamatergic neurons, have an important role in long-term potentiation and long-term depression (24). As a result, endocannabinoid signaling is a dynamic and localized process that maintains and prunes neuronal connections and provides a buffer in opposition to brain stress systems. When consumed, THC enters the brain globally and can interfere with this coordinated signaling by indiscriminately binding to CB_1_ receptors throughout the brain, modulating other neurotransmitters' release, and affecting stress responsiveness. In turn, enhanced stress reactivity can lead to the development of aversive emotional states because of the overactivation of stress and anti-reward systems or the under activation of the anti-stress systems (1, 25). Thus, after chronic use, the absence of THC may unmask this enhanced stress response, manifesting as withdrawal symptoms, contribute to the development of negative reinforcement, and be mitigated by substance use relapse.

Currently, there are no FDA approved medications for the treatment of CUD. Medications that have been shown to be effective for other SUDs, including naltrexone (alcohol and opioid use disorders) and bupropion (tobacco use disorder), or have demonstrated promise, such as topiramate (alcohol and cocaine use disorders), were without success (2). Trials of medications aimed at reducing cannabis use or cannabis withdrawal symptoms, including antipsychotics, antidepressants, and mood stabilizers among several others, have also been largely ineffective. To date, treatments demonstrating the most promise in clinical studies are cannabinoid partial agonists (THC, nabiximols). However, significant barriers related to cost and access in addition to their abuse potential make these currently impractical strategies. *N*-acetylcysteine, which has been shown to reduce symptoms of obsessive-compulsive behavior, has shown promise in studies of adolescents, but not adults, with CUD. Some medications that, broadly speaking, have sedating or anxiolytic potential, namely zolpidem, gabapentin, and quetiapine, have demonstrated some potential for reducing cannabis withdrawal symptoms (26). The identification of an effective pharmacotherapy for CUD remains a critical unmet need.

While cannabis has undergone a renaissance and expansion in its use for a variety of neuropsychiatric disorders, psychedelics have similarly been recently reconsidered and undergone an explosion of interest for treating psychiatric disorders and SUDs. Promising research in the 1950's through 1970's showed potential in the therapeutic use of hallucinogens in the treatment of alcohol and opioid dependence, before psychedelic research was largely extinguished until the last decade (27). During that time, studies provided preliminary data on the safety and feasibility of psychedelic use in treatment of SUD and smoking cessation (28), but assessment of psychedelics in the treatment of CUD has largely remained unexplored.

To date, there have been no clinical trials reported on the feasibility of using classical psychedelics for CUD. Intriguingly, an online survey by Garcia-Romeu et al. has suggested that psychedelics might be related to reductions in cannabis use (among other substances)−444 individuals who have used psychedelics in a non-clinical setting retrospectively reported reductions in cannabis use. These individuals reported lasting reductions for over a year after using a psychedelic. Although this study was limited by the nature of the anonymous, retrospective self-report data, and cannot be verified, it shows curious promise in the potential use of psychedelics as a treatment for CUD (29). This prompted us to explore the potential of classical (e.g., psilocybin, lysergic acid diethamide [LSD]) and non-classical (e.g., 3,4-methylenedioxymethamphetamine [MDMA], ketamine) psychedelics for treatment of CUD based on existing available clinical data, rational psychopharmacology, hallucinogenic and/or psychological experiences, and safety considerations.

We sought to answer the question: Do psychedelics have therapeutic potential for CUD? To address this question, we assessed the published literature for studies or reports that examined individuals with or at risk for CUD, who were administered or took psychedelic substances, and were observed for any change in cannabis use characterization or risks associated with CUD. Given the dearth of available research and data on this topic, we furthered our inquiry to consider: Is there psychopharmacological rationale for psychedelics in treatment of CUD? If psychedelics were to be used in treatment of CUD, what safety considerations would need to be taken into account? We utilized background sources identified in our search and supplemental literature to address the questions of rationale and safety.

## Methods

We conducted a search for published clinical trials in PubMed and other databases including Cochrane and EBSCOhost by combining the terms: “Cannabis use disorder” or (“cannabis or marijuana” and “dependence or abuse or addiction”) and “psychedelic or psilocybin.” Given the limited results returned, a follow-up and inclusive search was conducted which: (1) changed clinical trials to human research; (2) expanded the terms in “psychedelic or psilocybin” to increase sensitivity for possible cases the MeSH term “psychedelic” insufficiently captured compounds previously reported in human research as ascertained in our background familiarization: “psychedelic or psychedelics or psilocybin or MDMA or DMT or ketamine or mescaline or LSD”; and (3) added a term to additionally and broadly capture compounds with a molecular mechanism common to most psychedelics (i.e., agonist activity at serotonin 5HT-2a receptors) in an effort to provide support for mechanistic inference and include compounds that are relevant (e.g., novel compounds under development) but potentially not identified as psychedelics: “or 5HT2 agonist.” The search strategy included filters for language (English), year of publication (1990-present), and study subjects (humans). We searched government sponsored clinical trial registries (clinicaltrials.gov, euclinicaltrials.eu, clinicaltrialsregister.eu, australianclinicaltrials.gov, and clinicaltrails.health.nz) using the term “cannabis use disorder” to identify any registered studies (both active and inactive) utilizing psychedelic compounds as an intervention.

Inclusion criteria: Primary research and reports in individuals with or at risk for CUD (e.g., individuals who use cannabis heavily or frequently, cannabis abuse, cannabis dependence), and in whom cannabis use and/or risks associated with CUD were reported before and after receipt of a psychedelic.

Exclusion criteria: Literature that included psychedelics and CUD that are without direct interaction (i.e., indirectly described in the clinical sample but not under study, and/or cannabis use or risks associated with CUD were not reported after psychedelic use or administration), observational or epidemiological studies evaluating polysubstance abuse that included cannabis and repeated or chronic use of psychedelics (e.g., cohort description of cannabis use in a study of those who use MDMA regularly). Reviews and basic science (e.g., animal studies) literature were utilized for background information and rationale development.

Review of studies for inclusion was carried out independently by two reviewers and disagreements were resolved by discussion. The authors/reviewers have no identifiable conflicts of interest to report. The same eligibility criteria were applied to the title and abstract screen as to the full-text screen. Supplemental background information was gathered from references of retrieved and reviewed works.

A risk of bias assessment was conducted using a modified Downs and Black checklist (30), in order to include both randomized and non-randomized studies, and assess for quality criteria encompassing reporting, external validity, internal validity, and power. Scoring modifications were made following precedent reports. For randomized studies, the full 27 item scale with a maximum score of 28 was used with studies classified as excellent (26–28), good (20–25), fair (15–19), and poor (≤ 14) quality (31). For non-randomized studies (e.g., the case report) 10 irrelevant questions were removed (those assessing blinding, loss to follow up, cohort selection, and power) yielding an adjusted maximum score of 18, with classifications of excellent (14–18), good (9–13), fair (4–8), and poor (< 4) quality (32).

## Results

**Figure 1:**
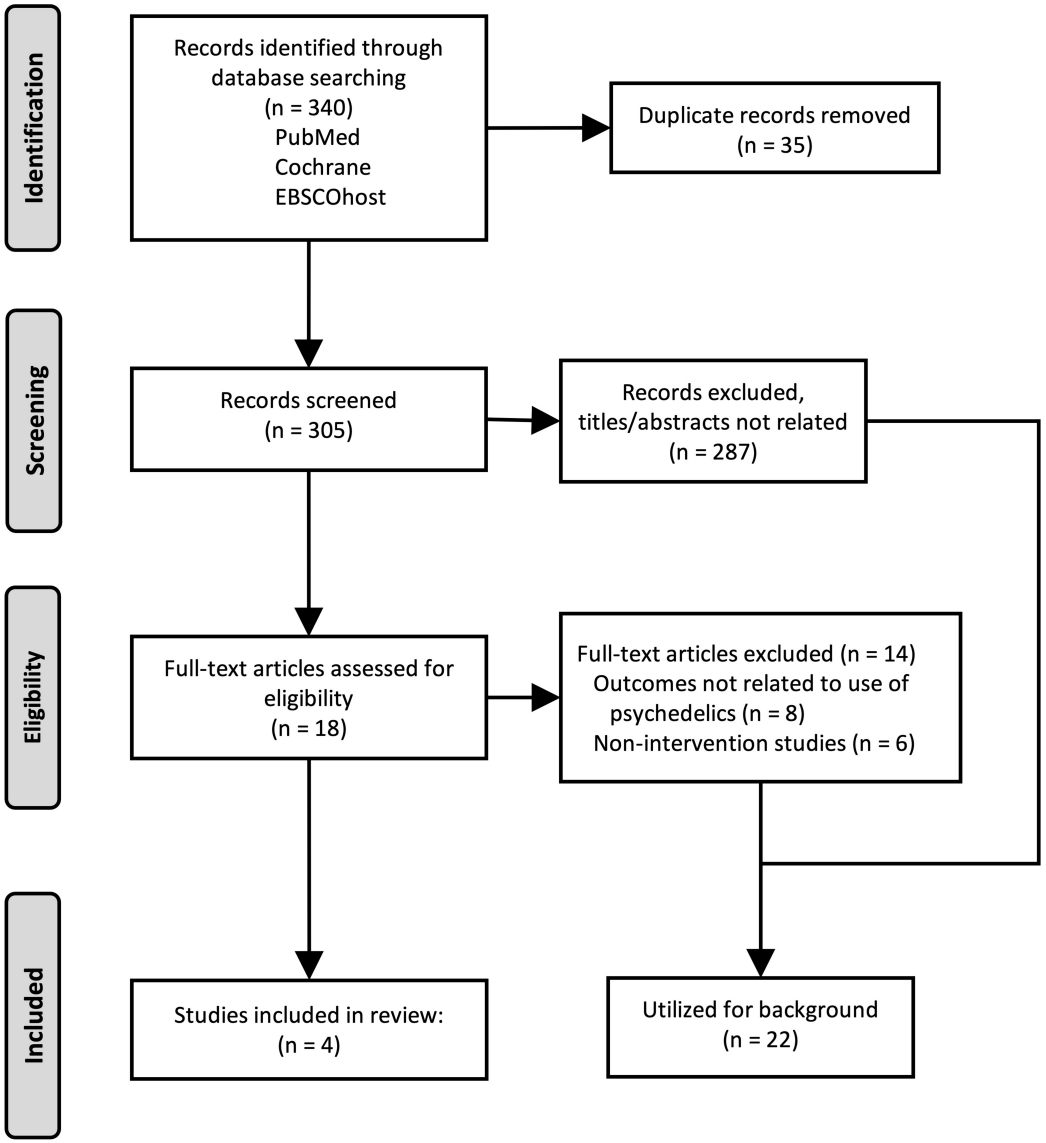
Flow diagram of the study selection strategy and results. Original research articles were identified and selected for inclusion as described, and are presented using the Preferred Reporting Items for Systematic reviews or Meta-Analyses (PRISMA).

Our searches were conducted on November 12, 2022 and December 12, 2022, and identified 305 unique results (Figure 1). No articles were identified that reported use of classical psychedelic substances for treatment of CUD. One article was identified that reported use of a non-classical psychedelic for CUD (ketamine). Further review revealed: one article examining therapeutic use of MDMA for PTSD which reported subsequent effects on alcohol and substance use (including mild CUD); one article examining therapeutic potential of a selective 5HT_2C_ agonist in non-treatment seeking individuals with daily cannabis use; and one case report of reduction from heavy cannabis use following an adverse effect of recreational use of psychedelics (Table 1). Studies were primarily excluded for wrong study design (*n* = 168; e.g., observational or other non-interventional study), study of a drug in persons with or at risk for CUD other than a psychedelic (*n* = 97), or study population other than persons using cannabis (*n* = 36). Full-text reviews identified studies excluded because psychedelic use was noted as a population characteristic but unrelated to the study outcome (*n* = 8), and studies that were non-intervention studies (e.g., epidemiological, health services research, self-report questionnaires). Twenty-two articles were identified as background articles that provided ancillary support for the review and rationale of the study topic.

**Table 1:** Summary of literature included for review.

	**Participant/ patient group**	**Study type**	**Intervention**	**Comparator**	**Outcome**	**Modified downs and black checklist score**	**Reference**
Azhari et al. (33)	Cannabis Dependence (*n* = 8)	Single blind, without placebo control	Ketamine 0.71 mg/kg or 1.41 mg/kg; paired with MET, MBRP	Ketamine one-vs. two-doses	85% reduction 7 days after 1^st^ infusion; 90% reduction during last 7 days of study; improved confidence in abstaining from cannabis; no change in cannabis craving	22/28 (Good)	(33)
Arout et al. (34)	Non-treatment seeking daily cannabis smokers (*n* = 15)	Placebo controlled, counter-balanced, within-subject human laboratory study	Lorcaserin 10 mg BID (5 HT_2C_ agonist)	Placebo	“Relapse” phase (3 days): Less cannabis use on days 1 & 2. ‘Abstinence Initiation' phase (3 days): Less cannabis use on day 1; less craving	23/28 (Good)	(34)
Nichol et al. (35)	Mild CUD (*n* = 2) within a larger study of PTSD participants (*n* = 82)	Double blind, randomized, placebo controlled trial	MDMA-assisted therapy (MDMA 80 + 40 mg 1^st^ session, 120 + 60 mg 2^nd^ and 3^rd^ sessions with 3-90 min therapy sessions each before and after MDMA sessions)	Placebo	Mild CUD participants both received placebo, therefore no outcomes available; No difference in change of DUDIT score pre-/post-sessions between overall PTSD MDMA/placebo groups	26/28 (Excellent)	(35)
Nutting et al. (36)	16-year-old female with history of CUD	Case Report	LSD (by self-report)	n/a	HPPD; Reduced cannabis use	7/18 (Fair)	(36)

BID, twice daily; CUD, cannabis use disorder; DUDIT, drug use disorder identification test; HPPD, hallucinogen-persisting perception disorder; LSD, lysergic acid diethamide; MBRP, mindfulness-based relapse prevention; MDMA, 3,4-methylenedioxymethamphetamine; MET, motivational enhancement therapy.

### Human studies of psychedelics or related compounds in CUD

Although classical psychedelics have not been directly studied in clinical trials to treat CUD, non-classical psychedelics and agents with related mechanism of action have shown promise of decreasing cannabis use in a small number of participants in clinical trials which support future investigation of psychedelics for CUD. Clinical case reports, while not quantitative and potentially prone to bias, can be informative during the foundational stages of research, and is included for review here.

#### Ketamine

Ketamine is an NMDA receptor antagonist and is variably referred to as a non-classical psychedelic or dissociative agent. At sub-anesthetic doses, ketamine has been shown to be potentially beneficial in alcohol and cocaine use disorders. A recent single-blind 6-week pilot study assessed the feasibility and tolerability of ketamine in eight participants with CUD. Participants received motivational enhancement therapy and mindfulness-based relapse prevention behavioral treatments in addition to a ketamine infusion of 0.71 mg/kg, with non-responders receiving a second infusion at 1.41 mg/kg. While there was no control group, compared to before treatment participants had a statistically significant reduction in cannabis use following treatment, and improvement in self-reported confidence in resisting the urge to use cannabis. The study additionally demonstrated the feasibility of integrating a psychedelic drug into behavioral treatment targeting CUD (33).

#### Lorcaserin

Lorcaserin is a selective 5HT_2C_ agonist which was briefly marketed as a weight loss medication. It has shown promise in tobacco use disorder, having demonstrated higher rates of tobacco use cessation (15.3%) compared to placebo (5.6%), but was tested without success in opioid and cocaine use disorders. In a study comprising of two 13-day human laboratory inpatient admissions, lorcaserin 10 mg BID was found to decrease cannabis self-administration in 15 non-treatment seeking individuals with daily use, compared to placebo. Lorcaserin also decreased craving during abstinence conditions, and co-morbid tobacco smokers decreased their tobacco use, even though they were not intending to (34).

5HT_2C_ agonists influence behaviors by a wide range of reinforcers including reducing the stimulating and reinforcing effects of nicotine, ethanol, opioids, and cocaine, likely by inhibiting mesocorticolimbic dopamine release. 5-HT_2C_ receptors overlap with dopaminergic and GABAergic receptors in brain areas relevant to drug seeking and reward, namely prefrontal cortex, ventral tegmental area, caudate putamen, and nucleus accumbens (37). They also improve inhibitory control which shows promise in reducing the likelihood of relapse. While not a psychedelic, lorcaserin is a selective agonist for arguably one of the more important neuroreceptor targets of classic psychedelics after 5HT_2A_ (38), and therefore may be of relevant consideration in dissecting the contribution of secondary targets in psychedelic therapeutics (39, 40).

#### MDMA

MDMA, a stimulant with mixed pharmacological effect which include the increase of oxytocin, has shown tremendous promise as a novel treatment for posttraumatic stress disorder (PTSD), and therefore has undergone increasing study and scrutiny (41). Given the higher prevalence of cannabis use and CUD in those with PTSD compared to the general population (42), it should be anticipated that MDMA could be therapeutically administered in persons with co-morbid disorders. In a secondary analysis of a study of MDMA for PTSD which allowed inclusion of participants with mild CUD or alcohol use disorder (AUD) or moderate CUD or AUD in early remission, only two participants were identified with CUD (both mild) who were randomized to the placebo group. Thirteen of the 21 participants with past, but not current, AUD received MDMA and had significant reductions in comparison to the placebo group of self-reported AUD and at-risk symptoms, which correlated to improvements in PTSD symptoms. In combination with the CUD participants, eight of the additional 14 participants with past, but not current, SUD received MDMA and had no significant change in self-reported SUD and at-risk symptoms (35). Therefore, no direct inferences regarding MDMA treatment in CUD can be made. While interpretations are hampered by the limited AUD and SUD severity and small size of the sample, that AUD and PTSD symptom improvements were correlated allows for speculation if co-morbid CUD could improve following successful treatment of PTSD with MDMA.

#### LSD: hallucinogen-persisting perception disorder and cannabis use

A case report of hallucinogen-persisting perception disorder (HPPD) in an individual with CUD provides an opportunity to consider the interaction and safety concerns of psychedelic use in those with CUD and comorbid disorders. A 16-year-old white female with a past psychiatric history of major depressive disorder, CUD, and social anxiety disorder tried LSD a total of four times over several months, each of which was a subjectively negative experience. Her first psychotic symptoms began following her initial use and she began to reexperience persisting symptoms of LSD intoxication immediately afterward. She had started with a low dose, and subsequent higher doses worsened her symptoms consisting of altered visual perceptions including tracers, trails, halos, and visual drifting. These episodes occurred numerous times daily and lasted seconds to minutes. Notably, she quit use of cannabis as it exacerbated her symptoms. She was admitted to a psychiatric inpatient unit 2 months after her last use of LSD following an intentional overdose of acetaminophen and ibuprofen. She had no contributory family history and her physical exam and labs, includeing urine drug screen, were unremarkable. She was diagnosed with HPPD, defined as episodic re-experiencing of hallucinations and mimicking acute hallucinogen intoxication following prior, but not recent, hallucinogen use; and specified as Type 2, which has more intense symptoms and more episodes with longer durations than Type 1, which is more self-limiting. Risperidone yielded significant improvement in her symptoms of psychosis but had no effect on those of HPPD (36).

This case is notable for the adverse effect of HPPD occurring after relatively few experiences with LSD, and the self-discontinuation of cannabis. While some or all of the LSD doses may have been above a range considered therapeutic, the number of reported experiences is close to that used in current clinical studies of other psychedelics (typically 1–3). While it remains unknown if this case represents an accelerated first break of an undeclared psychotic disorder, the reported history lacks clearly identifiable pre-morbid risk factors for psychotic disorders, and echoes long-identified concerns connecting LSD use to subsequent development of psychotic disorders (43–45). Of particular concern, regular cannabis use, which is well-described as an accelerant of psychotic disorders in adolescents and young adults, may have contributed to the negative outcome (46); to the extent that this concern can be extrapolated to other hallucinogens or other populations is unknown, but bears worth consideration. Finally, while HPPD is not an acceptable means to resolve CUD, the self-discontinuation of cannabis use in this case leaves a tantalizing contemplation if a more favorable outcome of reduced cannabis use without persisting psychosis could have been experienced if hallucinogens with lower doses or potency were used.

### Bias assessment

Bias and quality assessments of the clinical studies yielded good to excellent outcomes (Table 1). Studies were generally strong in essential reporting items, and variably limited by incomplete or lacking probability reporting, power analysis, blinding, randomization, and adjustment for confounding factors. Bias and quality assessment of the case report was fair. As a clinical report and not a hypothesis driven study, it was lacking in most of the data reporting pertinent to such studies.

## Discussion

Our search revealed a significant gap in the clinical research literature, as no organized studies assessing psychedelics in those with CUD have been reported. This limitation impacts both the effort to identify novel and needed treatments for CUD, and the understanding of safety considerations in utilizing psychedelic treatments in those with CUD or at risk for CUD, such as individuals who use cannabis heavily or regularly. As psychedelics are currently being studied for other SUDs, we have examined the literature to explore possible rationales for the mechanism, utility, and safety for their use in CUD.

Broadly, there are two dominant considerations for how psychedelics might be effective treatments for addiction: psychological changes often associated with mystical or spiritual experiences, and neuropharmacological actions. We will consider these two mechanisms in the context of CUD and SUDs, acknowledging that while they are worthy of individual study and consideration, they are likely not independent of each other. We will then consider anticipated safety concerns in administering psychedelics to individuals with CUD or regular heavy cannabis use, and provide recommendations for future studies.

### How might psychedelics be effective in addiction treatment, including CUD?

#### A profound experience may initiate changes in insight and self-efficacy

Psychedelic compounds can contribute to lasting effects in an individual within psychological domains such as mystical experience, mood and affect, and personality. Lasting behavior change can be triggered by an experience that is vivid, benevolent, mystical, and/or characterized by important insights. These “mystical experiences” are thought to provide profound alterations in perception along with a sense of meaningfulness, insightfulness, and unity. This state, achieved with support of psychedelics, is thought to be more malleable, flexible, sensitive to the environment, and open to change. Such experiences have also been reported with non-classic psychedelics, including “dissociative” compounds such as ketamine, or the “entactogen” MDMA. The therapeutic effects from these experiences can be enduring, and it is conceptualized that during the psychedelic experience a therapeutic window in the mind is temporarily opened which facilitates gained insight and emotional release. In conjunction with psychotherapeutic support, this insight can potentially lead to a healthy revision of outlook and lifestyle (47).

Psychedelics provide enhanced self-efficacy and increased motivation toward substance use reduction or cessation. This effect shows promise as a mechanism in sustaining abstinence, as was illustrated by a study in which participants given psychedelics who rated strong changes in self-efficacy and altered life priorities also had reduced tobacco use (48). In the study, participants were provided questionnaires designed to measure changes in attitude, moods, behavior, and spiritual experience during and after psilocybin treatment sessions, and were then asked to explain how they believed psilocybin had helped with their smoking cessation. Many of the responses had themes of self-motivation, changes in priorities, and insight. In a related consideration, persons using cannabis report reasons for initiating or achieving cessation of cannabis as related to changes in: self-image or self-control, emotional maturation, taking on new roles and responsibilities, health or legal concerns, and social relationship concerns (49, 50). Thus, it is conceivable that the enhancing self-efficacy through use of psychedelics could support a focusing of motivations to reduce or quit use of cannabis.

Generally, spiritual awakening, defined by “life change amounting to a new state of consciousness or being through the grace of a higher power, and leading to a new capacity for honesty, tolerance, unselfishness, peace of mind, and love,” has been noted as a significant predictor of substance use abstinence. This concept has been used in Alcoholics Anonymous and other 12 step groups (47). For a subset of those using cannabis, its use is spiritually motivated and achieves a similar effect to that of psychedelics in several experiences. Individuals who use cannabis and are spiritually motivated report greater insight into themselves and others, connection with nature and other people, greater love, joy, and feelings of disembodiment than non-spiritual individuals who use cannabis, and similar to that of experiences with psychedelics (51, 52). Notably, among spiritually motivated persons using cannabis, those without CUD compared to those with CUD of any severity reported less experiential avoidance, psychological distress, and used cannabis less often for coping or social reasons (53). Therefore, factors associated with a spiritual motive or experience, even in the absence of psychedelics, may be associated with positive outcomes potentially achievable with psychedelics, which in turn could reduce risks for or associated with CUD. This also supports the notion that the spiritual or mystical experience of psychedelics is likely a strong, but possibly insufficient, mediator of its mechanism of action.

#### Neuroreceptors targeted by, and neuroadaptations following, psychedelics

Psychedelics comprise several classes of drugs with mechanisms including 5HT_2A_ agonists, N-methyl-D-aspartate (NMDA) antagonists, kappa opioid receptor agonists, and muscarinic acetylcholine receptor antagonism, and which may bind to various other neuroreceptors, notably other members of the 5HT_2_ subfamily and 5HT_1A_. Classic psychedelics (e.g., psilocybin, LSD), which are also all essentially potent hallucinogens, act primarily as agonists at 5HT_2A_ receptors resulting in activation of cortical pyramidal neurons and downstream glutamate release. This is supported by demonstration that ketanserin, a 5HT_2A_ antagonist, can block hallucinations and most other subjective effects resulting from psilocybin (54). Classic psychedelics may also have lesser secondary effects via dopamine and other systems, as exemplified by haloperidol (a potent antagonist of D_2_, D_3_, and D_4_ receptors) achieving a 30% decrease of euphoria and depersonalization effects following psilocybin. However, haloperidol also increased the psychomimetic effect of psilocybin, suggesting that dopamine is not the primary mediator of these effects (47).

The serotonergic system has been targeted with the goal of treating specific symptoms of CUD and cannabis withdrawal, notably anxiety. This was partially the rationale behind trials using the 5HT_1A_ agonist buspirone and serotonin reuptake inhibitors as potential treatments for CUD (26). Relatedly, cannabidiol, which can temper some effects of THC, likely mediates some of its anxiolytic, antidepressant, and antiepileptic properties via 5HT_1A_ receptors (55, 56). In rats, the potent synthetic CB_1_/CB_2_ receptor agonist CP 55,940 caused a selective upregulation of 5HT_2A_ receptors in prefrontal cortex via a cascade of CB_2_ binding, increased phospholipase C β activity, and increased ERK1/2 activation (57). In parallel, chronic administration of CP 55,940 led to downregulation of CB_1_ and CB_2_ receptors, consistent with imaging results in human with chronic cannabis use (58). In addition to providing links between development of psychosis and anxiety following cannabis use, this cannabinoid-induced upregulation of 5HT_2A_ receptors provides a tantalizing link between the effects of chronic cannabis use and an essential target of psychedelic hallucinogens. As described in a retrospective observational study by Cox et al., psychedelics that target 5HT_2A_ receptors appear to reduce the consumption of cannabis (28). Furthermore, when 5HT_2A_ receptors are directly acted upon, they appear to enhance psychological domains noted above, such as insight, self-efficacy, and spirituality, suggesting potential for targeting serotonergic receptors for CUD treatment (29).

In contrast, quetiapine, an atypical antipsychotic and 5HT_2A_ antagonist, demonstrated in a 12-week randomized double-blind placebo-controlled trial of CUD participants an increased likelihood of transitioning from heavy frequency cannabis use to moderate use, and 10% reduction of cannabis withdrawal symptoms consistent with quetiapine's known effects on sedation and appetite. There was no reduction in cannabis use in light users, and there was no effect on abstinence, possibly due to insufficient motivation in the participants, and no effect on craving (59); however, in a prior human laboratory study, quetiapine was associated with an increase of craving in CUD participants (60), suggesting 5HT_2A_ antagonism has either neutral or adverse effect on cannabis craving.

Given that psychedelics have demonstrated a lasting therapeutic effect on SUDs, it is reasonable to consider if psychedelics result in persisting changes in the brain. On a molecular level, animal studies have demonstrated that stimulation of 5HT_2A_ receptors using the psychedelic 2,5-dimethoxy-4-iodoamphetamine (DOI) resulted in changes in synaptic strength in parietal cortex, other neocortical regions, and hippocampus. Animal models have also demonstrated that several psychedelics can stimulate neurogenesis and induce plasticity-promoting gene expression including brain derived neurotrophic factor (BDNF), while human studies using LSD have shown dose-dependent elevation of peripheral BDNF that correlated with psychoactive symptoms (61). In contrast, research participants with chronic cannabis use have been shown to have hippocampal volume reduction, which is partially reversible by cannabis cessation or cannabidiol augmentation (62, 63), and reduced synaptic density compared to cannabis non-users (64). Whether synaptic density could be strengthened or augmented by psychedelics in persons who use cannabis remains an unanswered question.

Persisting benefits could be mediated by pharmacologically assisted changes in brain connectivity. For instance, administration of LSD or psilocybin to healthy individuals led to increased openness which is a trait that is associated with cognitive flexibility, and 5HT_2A_ receptor agonism increases flexible and “divergent” thinking. It is suspected that psychedelics dysregulate activity in systems encoding inflexible thoughts and behaviors found in those with affective disorders and addiction. Resting state and functional connectivity studies have shown the default mode network (DMN), active in self-directed thought and introspection and inactive during mental tasks, is inappropriately maintained following THC exposure (65), while psychedelics lead to acute disruption within the DMN and strengthen connections to other networks. It has been proposed that this temporary desynchronization of the DMN disrupts top-down cognitive control (i.e., disrupting one's schema and experience-based interpretations), enhancing bottom-up processing (i.e., sensory focused, present-centered) which, while temporarily dissolving the boundary of internal and externally generated stimuli, also facilitates reassignment of prior beliefs and cognitive flexibility which can be enduring for up to 1 month (66). Therefore, this brief period can provide a critical window when new insights and associations can be developed, which could be enhanced with the support and reinforcement of a therapist. Furthering the therapeutic bond, psychedelics such as LSD and MDMA increase oxytocin, which may strengthen bonding experiences. This was exemplified by volunteers given LSD who reported significantly increased feelings of closeness, openness, trust, and happiness in the subjects (67).

### Safety and risks

Given the partially overlapping adverse potential of cannabis and psychedelics, risks common to the two substances, and risks elevated in persons with chronic cannabis use, should be considered.

Broadly, the psychedelics discussed hitherto are regarded as generally having a wide therapeutic index and favorable safety profile when administered in doses within the therapeutic range under controlled and comfortable settings. The most common adverse effects include anxiety, dysphoria, fear, confusion, increased blood pressure and heart rate, headache, nausea, fatigue, and dizziness which are typically dose-dependent and regarded as well-tolerated. Specific adverse effects of illusions (LSD), dissociation and sedation (ketamine), muscle tightening and jaw clenching (MDMA), and emesis (ayahuasca) are more compound specific, and similarly regarded as well-tolerated or essential to the therapeutic effect (67, 68).

Acute and chronic psychosis is an adverse effect of particular concern, as exemplified in the case study reviewed above. A systematic review by Studerus et al. including psychedelic-assisted therapy studies from 1999 to 2008 noted that among participants receiving psilocybin, 27% experienced fear and 17% paranoia. In other studies, 7% of subjects in the highest dose conditions fit the criteria for acute psychotic reactions. These events were confined to the acute phase and were managed by interpersonal support. Prolonged adverse effects of hallucinogen use such as psychosis and depression are found to be “exceedingly rare” in experimental settings. In another review, no incidences of prolonged psychotic reactions or precipitations or schizophrenia spectrum disorders were identified out of 110 subjects. However, one experienced symptom of emotional instability, anxiety, and depression which lasted for several weeks. A few subjects described mood swings, “excessive pensiveness and introversion” and memory/concentration issues after the drug session, which generally resolved after a few weeks (69). The risk of HPPD, as illustrated in the case report, is considered rare and the incidence incompletely known. While the use of psychedelics at therapeutic doses in supportive environments decreases the risk for acute or prolonged psychosis, the added vulnerability for psychosis in those with chronic cannabis use should add a layer of caution.

If provided as a treatment, it could be anticipated that individuals with CUD would have recently used cannabis or retain residual cannabinoids during psychedelic administration. The drug-drug interactions of THC, CBD, and psychedelics are unknown and likely multifaceted. However, their potential interaction on serotonin receptors (e.g., 5HT_1A_ where CBD acts as a partial agonist and several psychedelics as an agonist) and the overlapping sympathomimetic effects of each of them prompt consideration for potential negative responses. A survey study conducted by Kuc et al. evaluated the effects of cannabis use on the subjective quality of the psychedelic experience were investigated. Co-use of cannabis and psychedelics tended to be associated with more intense psychedelic effects, suggesting a synergistic effect between the two. High amounts of cannabis were associated with greater levels of fear and feeling of insanity, while low or medium cannabis co-use was scored less or not significantly different in those domains as well as paranoia, grief, and physical distress compared to no cannabis use (70). Similarly, cannabis withdrawal symptoms which include anxiety, irritability, insomnia, nausea, and physical discomfort could predictably contribute to adverse synergistic effects.

Cardiovascular disease has been associated with cannabis use whether through recreational use or for medicinal intentions. Complications can include acute coronary syndrome, cardiac arrhythmias, stroke, peripheral arteriopathy, stress cardiomyopathy, and sudden death. The mechanisms connecting cardiovascular risk and cannabis use are likely complex and are likely due to interactions between the endocannabinoid and autonomic nervous systems, specifically increased sympathetic tone and decreased parasympathetic tone (17). Both psychedelics and THC can produce a sustained increased heart rate and vasospasms and have procoagulant effects. Therefore, the co-use of cannabis and psychedelics may compound cardiovascular risks, and their use either independently or combined would be relatively contraindicated in persons with elevated cardiovascular risk or underlying disease.

Regarding the risk of addiction, development of a psychedelic use disorder is relatively low among recreational users, and in therapeutic studies using psilocybin there have been no reports of increased subsequent use of that or any other illicit drug (71).

### Strengths and limitations

Our search strategy was strengthened by including review of studies selected from three research databases, namely PubMed, Cochrane, and EBSCOhost, and screening for the best level of evidence available for our question (i.e., randomized human trials). However, the limited results available to date prompted us to expand the depth of the search strategy by including a follow-up collection of records using search terms less directly identified as psychedelics (5HT2 agonists). The recovered literature permitted inferences based on reported mechanism and experience with psychedelics in clinical settings and preclinical research.

Our study is limited by the search strategy's focus on clinical research, which likely contributed to the exclusion of potentially relevant articles based on self-report surveys (e.g., Kuc et al., Garcia-Romeu et al.). The search strategy did not capture potentially relevant literature on the basic science of psychedelic pharmacology and its intersection with mechanisms of CUD, which could provide helpful background information for the present study or deepen the understanding of potential mechanisms for the results observed among included studies. Additionally, our search did not include literature focused on individuals who use MDMA heavily and also use cannabis because of the lack of relevance to the study. However, future evaluation of this population might provide insight into comorbid use and potential complications of MDMA and cannabis co-use.

## Conclusion and recommendations

Research in psychedelics and clinical implementation of psychedelic-assisted therapy for treatment of SUDs and psychiatric disorders is gaining interest and attention both within and beyond the medical field. Despite the significant and growing prevalence of CUD, the need for novel therapies for CUD, and the high co-morbidity of CUD with psychiatric disorders for which psychedelics are being actively investigated, there is a substantial lack of research and understanding on the potential benefits or risks of psychedelic in treatment of CUD. As restrictions to cannabis access continue to decrease and psychedelics are on the eve of a similar pattern of patchwork legalization, it is foreseeable that individuals with CUD or heavy cannabis use will undertake psychedelic treatments whether for the intention of treating CUD, treating a comorbid condition, or for exploratory and recreational use. Therefore, at minimum, given their known and shared serious risks of psychosis, development of psychotic disorder, and cardiovascular events, research of these potential adverse outcomes with psychedelic use in persons who use cannabis should be conducted.

Furthermore, it is recommended that safety trials in persons with mild to moderate CUD without comorbid unstable illness be conducted prior to any treatment or efficacy trials. Though the proportion of individuals who have used psychedelics and cannabis is likely substantial and the report of serious adverse interactions is infrequent, that absence of data does not provide reassurance of safety. To date, psychedelic clinical trials in the modern age have largely included persons in good general health, and excluded those with severe or complex mental illness, family history of psychotic disorders, and important to this review, substance use disorders; future safety studies in the CUD population should include similar inclusion/exclusion criteria for consistency and to remain within the known safety parameters. As heavy and high potent cannabis use is more strongly correlated with adverse effects of concern to psychedelic use (namely, psychosis and cardiovascular effects), individuals with severe CUD are not recommended for inclusion until safety outcomes can be addressed in the CUD population. In addition, as examined by Kuc et al., heavy cannabis use was associated with adverse symptoms during psychedelic experiences. Since cannabis amount is subjectively variable, it may be challenging to individually identify those who have used a heavy amount. Therefore, it is recommended that CUD participants refrain from cannabis use prior to a psychedelic session to reduce the risk of psychological and cardiovascular adverse events, and not be experiencing withdrawal symptoms that could interfere with intended benefits or increase the risk for adverse effects.

Finally, given the potential of psychedelics in treatment of SUDs, critical information about the relative benefits, in addition to the risks, for persons with CUD or heavy cannabis use are needed to allow for informed decision making. While self-report surveys and the very limited clinical data gathered in this review are intriguing, the promise of psychedelics in CUD is largely cast from the cold beacon of hope (72) and renewed glow of their success in addressing AUD and other SUDs. Organized and prospective clinical studies should be conducted to objectively assess these novel treatments in CUD for possible efficacy and identification of participant or treatment parameters associated with success or failure in relevant outcome measures. As prior psychedelic studies have shown that mechanisms are likely multi-dimensional and the beneficial effects may last longer than conventional therapies, studies might be designed to assess neuroreceptor target engagement, changes in brain connectivity and plasticity, mystical or spiritual experiences, psychological changes that are of long-lasting duration, and treatment settings. In order to add to the general understanding of psychedelic therapies and provide coherent data, it is recommended that rating scales common to other psychedelic studies be implemented (e.g., 5-ASCD, Mystical Experience Questionnaire). Like other psychedelic studies, it is recommended that CUD treatment studies implement a therapist-assisted design. It is known that continued engagement in behavioral therapies is key to achieving cannabis use reduction or cessation, and psychedelics have the potential to strengthen a trusting bond between the patient and their therapist, which may increase compliance with treatment. As a mystical and spiritual component has been shown to be an important element to psychedelic therapy and suggested as a protective factor for CUD, the availability (without requirement) of spiritual guidance, rituals, or artifacts is recommended as a component of the therapeutic environment.

Despite the exuberance of the current psychedelic era which has launched a rush of scientific, clinical, and financial interest in the ushering of these treatments to the public, their application to specific disorders should be provided forethought and care. In the treatment of CUD, the use of psychedelics may have tremendous potential, and have predictable, and possibly unpredictable, risks.

## Data availability statement

The original contributions presented in the study are included in the article/supplementary material, further inquiries can be directed to the corresponding author.

## Author contributions

AP and GT contributed to the conception, search, review, analysis, and writing of the manuscript. All authors contributed to manuscript revision, read, and approved the submitted version.
